# A Drug Combination Rescues Frataxin-Dependent Neural and Cardiac Pathophysiology in FA Models

**DOI:** 10.3389/fmolb.2022.830650

**Published:** 2022-05-19

**Authors:** Rosella Abeti, Mittal Jasoliya, Sahar Al-Mahdawi, Mark Pook, Cristina Gonzalez-Robles, Chun Kiu Hui, Gino Cortopassi, Paola Giunti

**Affiliations:** ^1^ Ataxia Centre, Department of Clinical and Movement Neurosciences, UCL, Institute of Neurology, London, United Kingdom; ^2^ Department of Molecular Biosciences, School of Veterinary Medicine, UC Davis, Davis, CA, United States; ^3^ Department of Life Sciences, Institute of Environment, Health, and Societies, College of Health and Life Sciences, Division of Biosciences, Brunel University London, Uxbridge, United Kingdom

**Keywords:** Friedreich’s Ataxia (FA), Frataxin (FXN), Dimethyl fumarate (DMF), Resveratrol (Resv), Mitochondrial membrane potential (ΔΨ_m_), Reactive Oxygen species (ROS)

## Abstract

Friedreich’s ataxia (FA) is an inherited multisystemic neuro- and cardio-degenerative disorder. Seventy-four clinical trials are listed for FA (including past and present), but none are considered FDA/EMA-approved therapy. To date, FA therapeutic strategies have focused along two main lines using a single-drug approach: a) increasing frataxin and b) enhancing downstream pathways, including antioxidant levels and mitochondrial function. Our novel strategy employed a combinatorial approach to screen approved compounds to determine if a combination of molecules provided an additive or synergistic benefit to FA cells and/or animal models. Eight single drug molecules were administered to FA fibroblast patient cells: nicotinamide riboside, hemin, betamethasone, resveratrol, epicatechin, histone deacetylase inhibitor 109, methylene blue, and dimethyl fumarate. We measured their individual ability to induce *FXN* transcription and mitochondrial biogenesis in patient cells. Single-drug testing highlighted that dimethyl fumarate and resveratrol increased these two parameters. In addition, the simultaneous administration of these two drugs was the most effective in terms of *FXN* mRNA and mitobiogenesis increase. Interestingly, this combination also improved mitochondrial functions and reduced reactive oxygen species in neurons and cardiomyocytes*.* Behavioral tests in an FA mouse model treated with dimethyl fumarate and resveratrol demonstrated improved rotarod performance. Our data suggest that dimethyl fumarate is effective as a single agent, and the addition of resveratrol provides further benefit in some assays without showing toxicity. Therefore, they could be a valuable combination to counteract FA pathophysiology. Further studies will help fully understand the potential of a combined therapeutic strategy in FA pathophysiology.

## Introduction

Friedreich’s ataxia (FA) is an inherited multisystemic degenerative disorder, with a prevalence of ∼1–2/50,000, and has a typical onset during puberty (Labuda et al., 2000), which is ultimately lethal and for which there is no FDA/EMA-approved therapy. Clinical trials are being conducted for the omaveloxolone that has been proved efficacious (Lynch et al., 2018; Lynch et al., 2021), and the nicotinamide data also looked promising (Libri et al., 2014), but approval has still not occurred, so new molecules and new strategies are warranted. FA has only one cause, the genetic reduction of frataxin; thus, small molecules, proteins, or gene therapy that increases frataxin expression has received particular interest. Although gene and protein therapy strategies have been received enthusiastically by the pharmaceutical and investment community, it is still unclear whether gene delivery will be efficient enough for internal neural and cardiac tissues to support recovery. Thus, small molecules that penetrate the brain and heart and increase frataxin expression, whose deficiency is the only cause of FA, are also interesting.

The *FXN* gene encodes the mitochondrial protein frataxin (FXN), which is fundamental in iron–sulfur cluster biogenesis and mitochondrial function (Tsai and Barondeau, 2010; Colin et al., 2013; Abeti et al., 2016). Several studies have shown that some compounds increase *FXN* mRNA levels in patients’ blood (Libri et al., 2014; Soragni and Gottesfeld, 2016), but it is unclear if the drugs efficiently increase FXN expression at a cellular level. Therefore, we chose a combinatorial approach to screen drugs that have been already proposed for FA and administered them to primary fibroblasts from FA patients. By measuring mtDNA and the levels of *FXN* mRNA, we determined the efficiency of eight compounds: nicotinamide riboside (NR) (Bieganowski and Brenner, 2004; Chan et al., 2013), hemin (He) (Napoli et al., 2007), betamethasone (BM) (Mason et al., 2010; Giardino et al., 2013), resveratrol (Resv) (Alcaín and Villalba, 2009; Haigis and Sinclair, 2010), epicatechin (EC) (Qureshi et al., 2021), HDAC109 (Soragni and Gottesfeld, 2016; Rodden et al., 2021), methylene blue (MB) (Bistas and Sanghavi, 2021), and dimethyl fumarate (DMF) (Sahdeo et al., 2014; Jasoliya et al., 20 17; Jasoliya et al., 2019). This pool of drugs has been shown to either increase *FXN* or ameliorate mitochondrial function. In brief, NR has been described as a natural NAD^+^ precursor (Bieganowski and Brenner, 2004). NAD^+^ regulates sirtuin (class III HDACs) function and, consequently, oxidative metabolism, and it is a rate-limiting co-substrate for sirtuin enzymes. This NAD^+^ precursor differs from nicotinamide mononucleotide (NMN) as it requires the conversion to NMN by nicotinamide riboside kinase 1 (NRK1) (Bieganowski and Brenner, 2004). Sirtuins are a class of proteins that possess deacetylase properties and are responsible for cellular regulation (Bieganowski and Brenner, 2004). Therefore, by inhibiting their natural function, it is possible to reverse *FXN* silencing (present in FA patients) (Chan et al., 2013). He (ferric chloride heme or hemin) is an iron-containing porphyrin with chlorine that can be formed from a heme group and was suggested to improve mitochondrial activity in FA models (Napoli et al., 2007). BM is a corticosteroid capable of crossing the blood–brain barrier (BBB) and reaching the CNS, where it seems to suppress inflammation and even possibly act as an antioxidant (Mason et al., 2010; Giardino et al., 2013).

Resv (3,5,4′-trihydroxy-*trans*-stilbene) is a stilbenoid, a type of natural phenol, which, according to *in vitro* studies, can activate sirtuin 1(Alcaín and Villalba, 2009). In turn, sirtuin 1 activates the transcriptional coactivator PGC-1α (Haigis and Sinclair, 2010). This factor is known to play a role in FA pathogenesis (Coppola et al., 2009; Marmolino and Acquaviva, 2009; Yiu et al., 2015). In addition, PGC-1α induces mitochondrial biogenesis and antioxidant defenses (Marmolino et al., 2010).

EC is a phenol with antioxidant properties that can cross the blood–brain barrier (BBB) and activate the brain-derived neurotrophic factor (BDNF) pathways. It has also been proposed as a treatment for FA, and its clinical trial is currently in phase 2 (Qureshi et al., 2021). HDACi109 is an HDAC inhibitor that, as Bidichandani and others have shown, is able to revert nucleosome occupancy at the *FXN* promoter and relieve the transcription initiation defect in FA patient's cells (Rodden et al., 2021). MB, methylthioninium chloride, is a salt used as a medication and dye. Its primary therapeutic use is to treat methemoglobinemia (Bistas and Sanghavi, 2021), but it has been found efficacious also on FA by promoting mitochondrial biogenesis and increasing *FXN* levels (Khdour et al., 2018). DMF is the methyl ester of fumaric acid known for its anti-inflammatory and cytoprotective properties (Linker et al., 2011). DMF is known to stimulate the activity of the transcription factor nuclear factor (erythroid-derived 2)-like 2 (Nrf2) (Scannevin et al., 2012; Chen et al., 2014). It has been previously identified during a 1600 FDA-approved drug library screening and subsequently proved to protect mitochondria in a dose-dependent cell-based assay in FA (Sahdeo et al., 2014). DMF enhances mitochondrial biogenesis (Jasoliya et al., 2017) and significantly increases *FXN* expression in FA cells by increasing transcription initiation (Jasoliya et al., 2019).

We preformed our assays commencing with a single drug administration, and then we combined some of them in pairs to see whether drug combinations could improve the efficacy of a single drug. These combinations were then tested for efficacy in cultures of primary cerebellar neurons and cardiomyocytes prepared from FA mouse models, looking at the mitochondrial function and mitochondrial reactive oxygen species (mROS). Then we have also tested the neurobehavioral effect in a mouse model of FA.

## Materials and Methods

### Primary Human Fibroblasts

Primary cultures of human control (GM-3440) and FA patient fibroblasts (GM-4078) were obtained from the Coriell Institute for Medical Research repository. They were maintained at 37°C in a humidified atmosphere with 5% CO_2_. DMEM (Corning, Inc., Corning, NY, United States) supplemented with 10% fetal bovine serum (JR-Scientific, Woodland, CA, United States) and 1x penicillin–streptomycin solution (Corning, Inc., Corning, NY, United States) was used as the growth medium. The medium was changed every 2 days (Jasoliya et al., 2017). Treatments with the drugs were performed 48 h prior to the assays.

### Transgenic Mice

All the transgenic mice were purchased from Jackson Laboratory and housed at UCL, except for the YG8LR mouse model that was from the Pook repository and housed at Brunel University. All procedures were carried out in accordance with the U.K. Home Office Animals (Scientific Procedures) Act 1986 (ASPA). The protocols were approved by the UCL Animal Welfare and Ethical Review Body (AWERB) and by the Brunel University Animals Welfare and Ethical Review Board. The KIKO model was originated by a frataxin knock-in/knockout (KIKO), with one allele of the frataxin (GAA)_230_ expansion mutation (*Fxn* ^*tm1Pand*^) on one chromosome and one allele of the frataxin exon 4-deleted mutation (*Fxn* ^*tm1Mkn*^) on the homologous chromosome) (Marmolino et al., 2010). The corresponding control line used was the wild type (WT; C57BL/6J). The YG8R-_800_ model, also called Fxn^null^:YG8s (GAA) _> 800_ mice, harbors a global null allele of mouse frataxin (*Fxn* ^*null*^) and the human *FXN* YAC transgene small repeat YG8s (contracted integration to a single copy of the human *FXN* gene (Anjomani Virmouni et al., 2015)) with >800 GAA trinucleotide sequence repeats). The control line used to compare this model was the Y47R, *Fxn* ^*null*^ allele and a single copy integration of the Y47 human *FXN* YAC transgene encoding human frataxin with normal-sized (GAA)_9_ repeats. The YG8LR model was originated by a mouse colony called YG8sR, which was derived while breeding the former YG8R (which initially contained expanded (GAA)_90–190_ repeats units) (Al-Mahdawi et al., 2006). The YG8sR line contained a single copy of the *FXN* YAC transgene and a single pure GAA repeat expansion mutation (which was formerly of (GAA)_120_ repeats in size in the founder mouse). The GAA repeat remains as a single unit upon transmission but exhibited both intergenerational and somatic variability in repeat size, and that is how the YG8LR was generated. YG8LR line has a single pure GAA repeat expansion mutation, but the number of repeats reached (GAA)_600_. For the preparation of primary cultures of cerebellar granule neurons and cardiomyocytes, we have used the KIKO mouse model. The YG8R-_800_ mouse model was also used for primary cultures of cerebellar granule neurons. YG8LR was used for mtDNA, frataxin mRNA, and behavioral studies.

### Cerebellar Granule Neurons

CGNs from KIKO and YG8R-_800_ mice and their controls, respectively, WT and Y47R, were isolated using the method described in Abeti et al. (2016) with few modifications. In brief, cerebella were isolated and homogenized, and dissociated with 0.25% trypsin-EDTA (Sigma Aldrich). CGNs were plated on glass coverslips, pre-coated with poly-D-lysine (1 mg/ml). The cells were maintained in Neurobasal with 2% of B27 (Invitrogen) and 2 mML-glutamine, 1% of penicillin/streptomycin (Sigma Aldrich), and 25 mM KCL (to keep the CGNs slightly depolarized). In addition, 10 µM AraC was added after 24 h from the plating to avoid glial proliferation. CGNs were used 7 days after plating. Cells were maintained in a humidified incubator at 37°C and 5% CO_2_. Prior to experiments, the cells were treated 48 h with the different compounds.

### Primary Cultures of Neonatal Cardiomyocytes

Hearts were isolated from 6-day-old KIKO and WT mice, and cardiomyocytes were prepared. In brief, the hearts were homogenized and digested with isolation buffer solution composed (millimolars): 116 NaCl, 20 HEPES, 0.77 NaH2PO, 5.5 D-glucose, 5.4 KCl, and 0.4 MgSO4, with pH adjusted to 7.35. In 100 ml isolation buffer, 60 mg collagenase type II CLS2 (∼250 µ/mg, Worthington) and 25 mg pancreatin from porcine pancreas (Sigma) were added. After digestion and cardiomyocyte extraction process, the cells were plated in Dulbecco’s modified Eagle medium supplemented with 15% fetal calf serum and 1% penicillin/streptomycin and maintained in an incubator at 37°C, 5% CO_2_^32^.

### Drugs

The concentrations for each molecule used in the study in primary cell cultures (human and mouse) and administered 48 h prior to experiments were as follows: NR (100 μM; reconstitute in H_2_O; #SMB00907); He (10 μM; reconstitute in DMSO; #51280); BM (10 μM; reconstitute in DMSO; #B7005); Resv (100 μM; reconstitute in DMSO; #R5010); EC (100 μM; reconstitute in DMSO; #E4018); HDAC109 (5 μM; reconstitute in DMSO; #382149); MB (3 μM; reconstitute in H_2_O; #319112); and DMF (30 μM; reconstitute in DMSO; #242926). For vehicle-treated cells, we used the highest concentration of the solvent used to reconstitute the compounds that was ≤0.1% (DMSO or H_2_O). All the drugs described before were purchased from Sigma-Aldrich. Caffeine (10 mM; reconstitute in H_2_O; #2793; Tocris Bioscience) was administered during live imaging experiments on cardiomyocytes.

### DNA and RNA Isolation and Quantification

Total DNA was extracted from primary fibroblast cells and mouse tissues using a DNeasy plus mini kit and DNeasy blood & tissue kit (Qiagen, Valencia, CA, United States) following the manufacturer’s instruction (Jasoliya et al., 2019). DNA was quantified by a NanoDrop 2000c Spectrophotometer (Thermo Scientific, Waltham, MA, United States). Total RNA was extracted from human fibroblast cells using an RNeasy plus mini kit (Qiagen, Valencia, CA, United States), following the manufacturer’s instructions. RNA quantity and quality were measured by using a NanoDrop 2000c Spectrophotometer (Thermo Scientific, Waltham, MA, United States). For mouse tissues, total RNA was isolated by homogenization with TRIzol (Invitrogen, United Kingdom).

### Quantitative PCR

cDNA, from primary fibroblasts, was synthesized from mRNA with iScript cDNA Synthesis Kit (Bio-Rad Laboratories, Hercules, CA, United States) per manufacturer’s instruction in a C1000 Touch Thermal Cycler (Bio-Rad Laboratories, Hercules, CA, United States). qPCR was performed using SensiFAST SYBR No-ROX Kit (Bioline, Taunton, MA, United States) in Roche Lightcycler 480 (Roche Diagnostics, Indianapolis, IN, United States). The second derivative of the amplification curve was used to determine the cycle threshold, and the data were analyzed by Δ_CT_ calculation. Primers used for mTL1 DNA for mitochondrial DNA and beta 2 microglobulin for nuclear DNA (*B2M*) and *FXN* are detailed in Table 1 (Jasoliya et al., 2017); (Jasoliya et al., 2019).

**TABLE 1:** Primers.

Species	Gene	Sequence (5′ → 3′)
Human	*MT-TL1* (DNA) Forward	CAC​CCA​AGA​ACA​GGG​TTT​GT
	*MT-TL1* (DNA) Reverse	TGG​CCA​TGG​GTA​TGT​TGT​TA
Human	*B2M* (DNA) Forward	TGC​TGT​CTC​CAT​GTT​TGA​TGT​ATC​T
	*B2M* (DNA) Reverse	TCT​CTG​CTC​CCC​ACC​TCT​AAG​T
Human	*FXN* Forward	ATC​TTC​TCC​ATC​CAG​TGG​ACC​T
	*FXN* Reverse	GCT​GGG​CAT​CAA​GCA​TCT​TTT

cDNA, from mouse tissues, was synthesized from mRNA, by using AMV reverse transcriptase (Invitrogen) with oligo(dT)20 primers, and FXN expression levels were determined by qRT-PCR using FRT-I and RRT-II primers as previously described (Anjomani Virmouni et al., 2015).

To determine the mt/nDNA ratio, total DNA was extracted from mouse brain tissue and qPCR analysis was performed using mouse mt-Nd1 and Cftr primers as previously described (Jasoliya et al., 2017).

### Mitochondrial Membrane Potential Assay

The maintenance of mitochondrial membrane potential (ΔΨ_m_) was assessed with tetramethylrhodamine methyl ester (TMRM; Invitrogen), a cationic fluorescent dye that enters healthy mitochondria. The dye was used with two different protocols: a) redistribution mode (Abeti et al., 2016). In healthy cells, at concentrations <50 nM, TMRM fluorescent signal shows a mitochondrial localization, which is retained until mitochondrial toxins induced depolarization. Upon ΔΨ_m_ depolarization, TMRM redistributes into the cytosol, and the signal decreases. The maintenance of ΔΨ_m_ was measured as the difference between the response to oligomycin (2 μg/ml) and the baseline. Then 1 μM rotenone (Rot) and 1 μM carbonyl cyanide-p-trifluoromethoxy phenylhydrazone (FCCP) were used to dissipate the ΔΨ_m_ completely. b) De-quenching mode (Abeti et al., 2018a): At concentration >500 nM, the dye goes to the mitochondria, but the vicinity of many fluorescent molecules together quenches the fluorescent signal. After that, 10 mM caffeine was used to challenge cardiomyocytes ΔΨ_m_. Upon depolarization, part of TMRM molecules exit the mitochondria, and the signal is de-quenched, showing an increase in fluorescence. TMRM was excited at 560 nm, and the images were collected with a 590-nm long-pass filter. Fluorescence was measured using a Zeiss 710 CLSM confocal microscope.

### Imaging Mitochondrial ROS

In total, 1 µM CM-H2Xros (Thermo scientific) was loaded for 20 min prior to the beginning of the experiments at RT and then imaged with 560-nm laser and long-pass 590-nm emission filter to assess mitochondrial ROS (mROS). Fluorescence was measured using a Zeiss 710 CLSM confocal microscope. CM-H2Xros is a non-fluorescent probe that accumulates into active mitochondria. Upon oxidation, the probe becomes fluorescent. The increase in fluorescence was analyzed within the mitochondrial region, and the rate was then calculated cell by cell and percentage performed (Abeti et al., 2016; Angelova et al., 2021).

### Behavioral Assays

Weight determination, beam walk, and rotarod performances were assessed monthly for a period of 5 months using the previously described methodology (Anjomani Virmouni et al., 2015). All procedures were carried out in accordance with the U.K. Home Office Animals Scientific Procedures Act (1986) and with ethical approval from the Brunel University London Animals Welfare and Ethical Review Body.

### Short-Term Studies

Groups of age- and sex-matched, 2- to 4-month-old, hemizygous YG8LR mice (*n* = 4–9) were formerly treated daily by intraperitoneal injection for 5 days with either vehicle (PBS/5% Tween 20/5% PEG 400/2% DMSO) or drug: (i) 10 mg/kg/d DMF, (ii) 10 mg/kg/d Resv, or (iii) 10 mg/kg/d DMF and 10 mg/kg/d Resv combined. Brain tissue was subsequently analyzed to determine the comparative levels of *FXN* mRNA expression and mt/nDNA ratio. The control vehicle group was only included as an example of the *FXN* expression and mtDNA levels. The level of *FXN* expression in YG8LR founder mice (YG8sR) compared with the Y47R mice has previously been reported (Anjomani Virmouni et al., 2015), and it showed that in brain tissue, the level of *FXN* is about 20% compared to control (Y47R) mice

### Long-Term Studies

Mice at 2–3 months of age were treated by oral gavage administration three times per week with either vehicle (PBS/5% Tween 20/5% PEG 400/2% DMSO) (*n* = 22) or 200 mg/kg/d DMF and 200 mg/kg/d Resv combined (*n* = 21). Monthly observations were made of mouse weights and coordination abilities, in both short- and long-term studies. Beam walk performance was examined by the time taken to walk across a 1-m-long, 22-mm-wide beam. The relative beam walk performance was determined by the average time to cross the beam during the test period compared to the initial pretreatment average time to cross the beam (seconds). Relative rotarod performance was determined by average latency to fall during the test period compared to the initial pretreatment average latency to fall (seconds). **p < 0.01 statistical significance obtained by Student’s t-test analysis of 5-month data.

### Statistical Analysis

Statistical analysis was performed with Excel, Origin 9 (Microcal Software Inc., ), and GraphPad 9 software (GraphPad Software, La Jolla, CA, United States). Results are expressed as means ± SEM or SDM. Student’s t-test and ANOVA tests were applied, when appropriate. The point of minimum acceptable statistical significance was taken to be 0.05 with Bonferroni correction, Tukey’s multiple comparisons test, and Dunnett’s multiple comparisons test. Representative averages were taken from *n* > 3 independent experiments.

## Results

### Drug Screening

**FIGURE 1:**
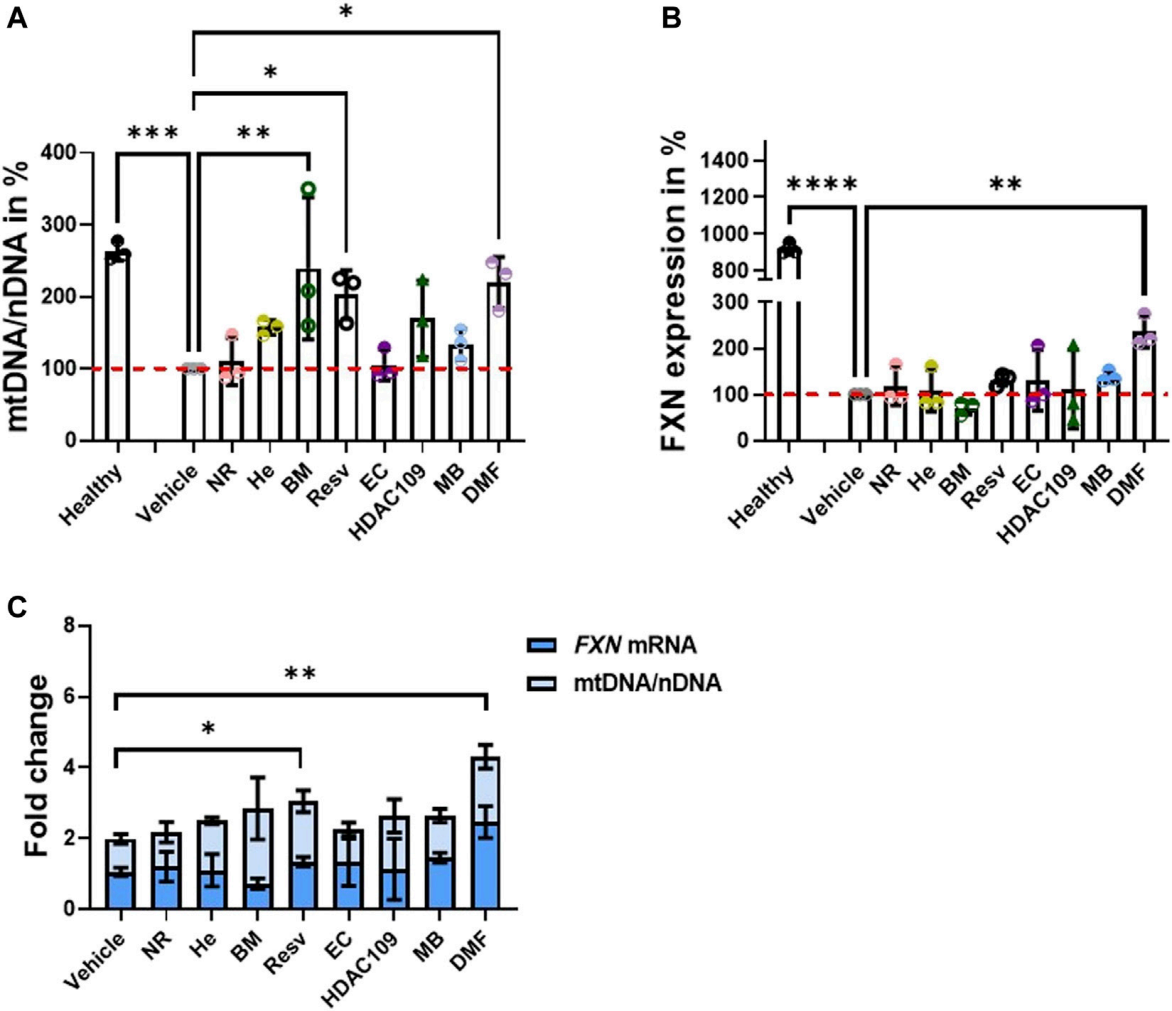
Testing the ability of drugs to promote mitochondrial biogenesis and *FXN* increase in the FA patient cell line. Forty-eight hours prior to cell harvesting, the different compounds were administered. **(A)** Histogram represents the levels of mtDNA/nDNA in percentages. Results were compared to vehicle (FA cells untreated) and showed that BM, Resv, and DMF increased significantly mtDNA (mean ± SD; healthy 263 ± 13, NR 109.9 ± 32.7, He 157.2 ± 10.21, BM 239.4 ± 98.86, Resv 202.6 ± 34, EC 105.1 ± 21, HDAC 109 169.6 ± 53, MB 133.9 ± 21.5, DMF 220 ± 35.3; one-way ANOVA with Dunnett’s multiple comparisons test). **(B)** Results were compared to vehicle (FA cells untreated); the histogram shows the *FXN* mRNA levels in percentage where most of the drugs showed an increase in expression, but DMF was the most efficacious. BM does not appear to increase *FXN* (mean ± SD; healthy 919.5 ± 30.4, NR 118.5 ± 41.45, He 108.9 ± 45.45, BM 71.3 ± 14.6, Resv 132.7 ± 13.1, EC 131 ± 66, HDAC 109 111.6 ± 85.3, MB 139 ± 12.6, DMF 236 ± 34.5; one-way ANOVA with Dunnett’s multiple comparisons test). **(C)** Plot combines *FXN* mRNA levels and mtDNA/nDNA ratio to visualize the fold changes of single dose (mean ± SD; vehicle 1.06 ± 0.12, NR 1.2 ± 0.42, He 1.1 ± 0.46, BM 0.72 ± 0.15, Resv 1.34 ± 0.13, EC 1.32 ± 0.7 HDAC 109 1.13 ± 0.9, MB 1.45 ± 0.14, DMF 2.46 ± 0.45; two-way ANOVA). ****p < 0.0001; ***p < 0.0005; **p < 0.005; *p < 0.05; *n* > 3 independent experiments).

We first tested primary FA patient fibroblasts for drug-based induction of mitochondrial biogenesis and *FXN* expression, which are both markers of FA pathology (Jasoliya et al., 2017; Jasoliya et al., 2019). The compounds were administered 48 h prior to the experiments, and mitochondrial DNA (mtDNA) copy number, instead of nuclear DNA (nDNA), was examined. We found that BM, Resv, and DMF showed a significant increase in this parameter (Figure 1A), thus proving that all three compounds promote mitochondrial biogenesis. These results were in line with previous reports in FA patient fibroblasts and PBMCs, and KIKO mice, which showed that DMF effectively increased mitochondrial biogenesis (Jasoliya et al., 2017). We then measured the *FXN* mRNA levels. By comparing the samples to the vehicle (FA-untreated cells), all the compounds, excluding BM, showed an increased mean of *FXN* mRNA expression, but only DMF-treated cells were statistically significant (Figure 1B). On the histogram, we have also reported that there is a remarkable difference between the healthy control and untreated and treated FA patients’ cells. This is because FA patients’ cells express frataxin at much lower levels (5–30% according to the GAA repeats length variability (Gellera et al., 2007), and it is acknowledged in the FA community that neither gene therapy nor small molecules approaches will be able to increase frataxin at healthy control levels. Reaching a closer level to carriers, which do not show any symptoms and their levels is around only 50% compared to control, it is likely to produce some benefit (Nachbauer et al., 2011; Saccà et al., 2011).

To visualize the effect of combining mtDNA increase to *FXN* mRNA levels, we plot the results together (Figure 1C). These results are in line with previous studies on FA models using DMF (Hui et al., 2021). The most promising candidates appear to be DMF and Resv as the changes induced by them were both statistically significant (Figure 1C). Consequently, we conducted physiological experiments using this combination.

### The Physiological Impact of Selected Compounds on Neuronal Cells

**FIGURE 2:**
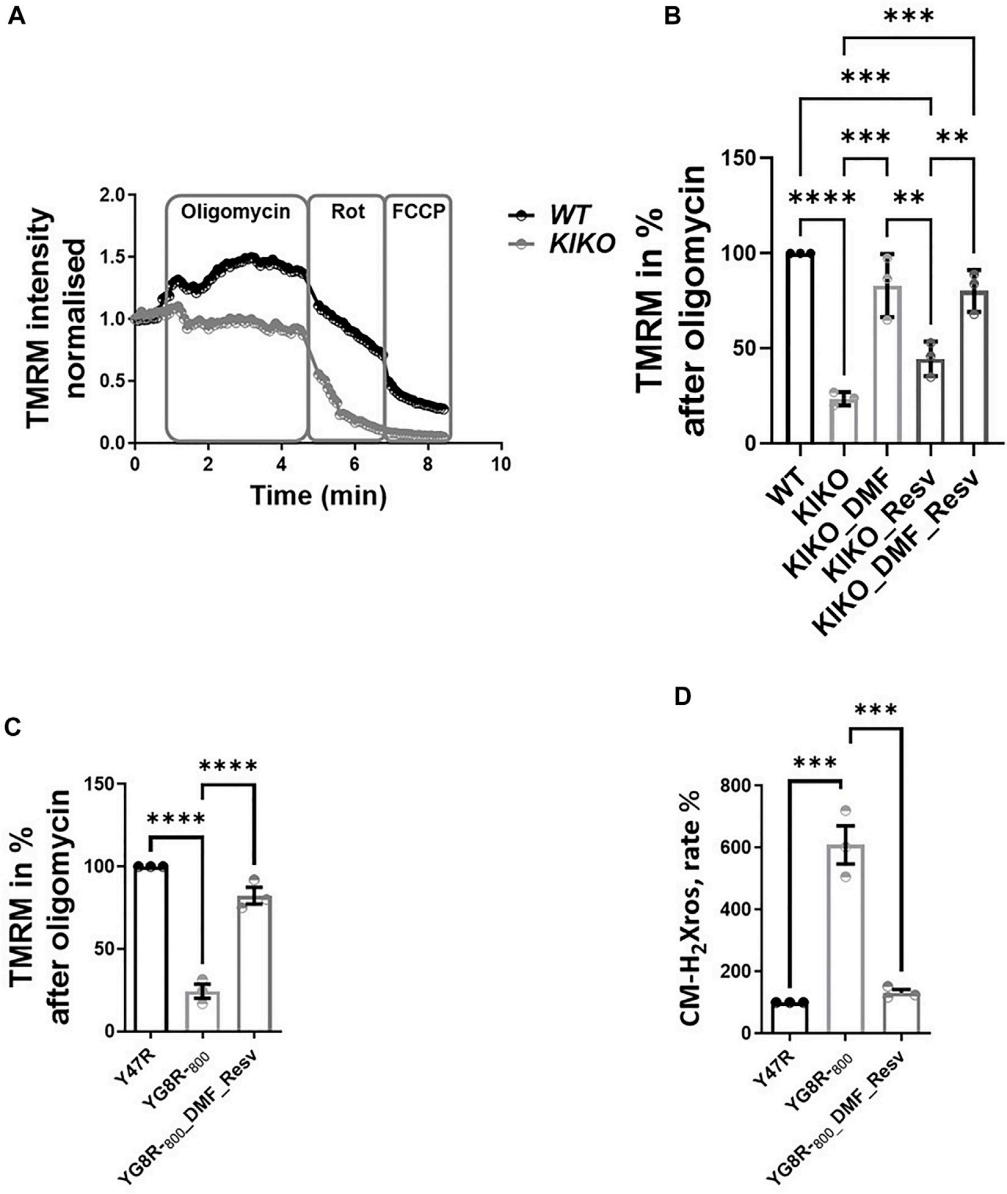
Efficacy of DMF and DMF and Resv to prevent depolarization in KIKO CGNs. Forty-eight hours prior to experiments, the different compounds were administered at the following concentrations: 30 µM DMF and 100 µM Resv. **(A)** Kinetic curves of the TMRM fluorescence assessing the ΔΨ_m_ challenged by 2 μg/ml oligomycin, 1 µM rotenone, and 1 µM FCCP. **(B)** Difference in ΔΨ_m_ response to oligomycin normalized to WT taken as 100% (KIKO 23.7 ± 3.5; KIKO_DMF 83 ± 16; KIKO_Resv 44.7 ± 9; KIKO_DMF_Resv 80.3 ± 10.9; ****p < 0.0001; ***p < 0.0005; **p < 0.005; *p < 0.05; *n* > 3 independent experiments). **(C)** Difference in ΔΨ_m_ response to oligomycin normalized to (control) taken as 100% (YG8R-_800_ 24.3 ± 7.4; YG8R-_800__DMF–Resv 82.4 ± 8.6). **(D)** Rate of mROS normalized to Y47R (control) (mean ± SD; YG8R-_800_ 609 ± 107; YG8R-_800__DMF–Resv 131 ± 19; one-way ANOVA with Tukey’s multiple comparisons test). ***p < 0.0005; *n* > 3 independent experiments.

We previously found that neuronal FA models showed mild mitochondrial bioenergetic deficiency (Abeti et al., 2016). Accordingly, we tested the efficacy of these drugs on primary cerebellar granular neurons (CGNs) from KIKO mice. Following incubation with 30 µM DMF, 100 µM Resv, and the combination of the two (DMF and Resv) for 48 h, we measured the maintenance of ΔΨ_m_, a readout for mitochondrial health (Figure 2). Figure 2 illustrates kinetic curves of TMRM fluorescence in CGNs used in the redistribution mode, in which upon depolarization, the fluorophore is released from the mitochondria and redistributed into the cell body, and the fluorescent signal decreases (Figure 2A). Upon administration of the ATP synthase (complex V) inhibitor oligomycin (2 μg/ml), the signal in WT neurons increased as expected because the electron transport chain (ETC) works harder to compensate the inhibition of complex V. However, under the same conditions, the signal in KIKO mice CGNs decreases, reflecting ΔΨ_m_ depolarization, indicating that the ETC is not able to maintain the potential. After Rot (1 μM) administration, the complex I-dependent electron transfer shows deeper depolarization. At the end of the experiment, the uncoupler FCCP fully dissipated the ΔΨ_m_ as expected (Figure 2A). The histogram summarizes the decrease in fluorescence after oligomycin administration (Figure 2B). Interestingly, DMF and the combination of DMF and Resv successfully prevented depolarization in KIKO CGNs, while Resv alone did not show a significant difference when compared to KIKO-untreated cells. This shows that Resv alone is unlikely to improve the imbalance in mitochondrial energy (Figure 2B). We then assessed ΔΨ_m_ and the mitochondrial reactive oxygen species (mROS) generation in CGNs (Figure 2C, D). Similar to KIKO CGNs, YG8R-_800_ cells displayed a marked ΔΨ_m_ depolarization, which was recovered by pretreating the cells with the DMF–Resv combination (Figure 2C). Last, several researchers have demonstrated that FA cells have decreased antioxidant protection (Chantrel-Groussard et al., 2001; Li et al., 2013; Abeti et al., 2018b), and this drug combination showed protection against mROS, which are otherwise toxic for YG8R-_800_ cells (Figure 2D).

### Physiological Impact of Selected Compounds on Cardiac Cells

**FIGURE 3:**
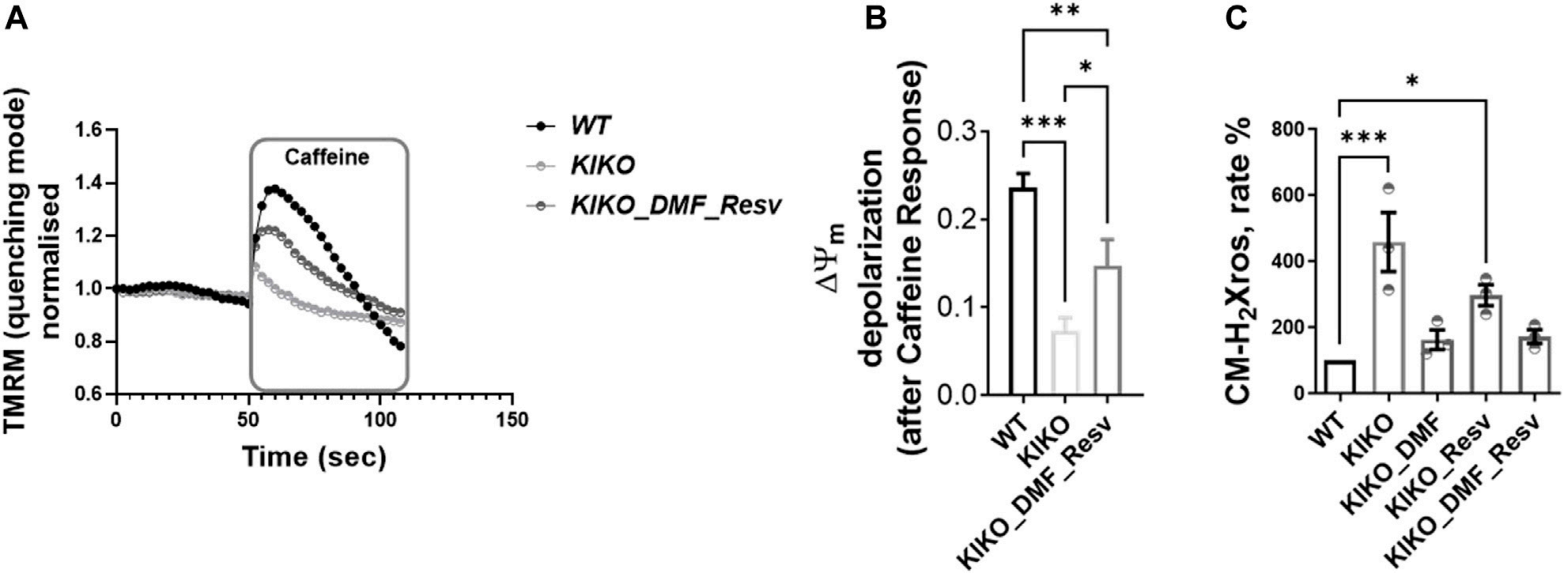
A-B. Physiological ΔΨm depolarization to caffeine restored with DMF–Resv. C. DMF–Resv prevents an mROS increase in cardiomyocytes. Forty-eight hours prior to experiments, the different compounds were administered at the following concentrations: 30 µM DMF and 100 µM Resv. **(A)** Kinetic curves of the ΔΨ_m_ response to caffeine. **(B)** Measure of ΔΨ_m_ depolarization after caffeine (mean ± SD; WT 0.23 ± 0.016; KIKO 0.07 ± 0.015; KIKO_DMF_Resv 0.15 ± 0.03; one-way ANOVA with Tukey’s multiple comparisons test). **(C)** Rate of mROS normalized to WT (mean ± SD; KIKO 460 ± 154; KIKO_DMF 163 ± 52; KIKO_Resv 297 ± 54; KIKO_DMF_Resv 172.4 ± 36; one-way ANOVA with Dunnett’s multiple comparisons test). ***p < 0.0005; **p < 0.005; *p < 0.05; *n* > 3 independent experiments.

Previously, we found that cardiac models of FA showed deregulation in Ca^2+^ homeostasis, in which the sarcoplasmic reticulum (SR) Ca^2+^ content was pathologically reduced, and that mitochondrial Ca^2+^ uptake was impaired (Abeti et al., 2018a). To test whether the DMF and Resv could effectively protect the cells from such aberrant response in cardiomyocytes, we have challenged the cells with caffeine, an agonist of ryanodine receptor (RyR)-mediated Ca^2+^ release. The increase in cytosolic Ca^2+^ then activates Ca^2+^ entry into the mitochondria, which stimulates the activity of the cardiac mitochondrial dehydrogenases, thus driving a change in ΔΨ_m_ (Abeti et al., 2018a). In Figure 3, the graph shows the ΔΨ_m_ depolarization in WT, which is the response to the events described before (here TMRM was used in the de-quenching mode; see Materials and Methods), that was much smaller in KIKO cells (Figure 3A). Interestingly, in DMF- and Resv KIKO-treated cells, the mitochondrial function was nearly restored (Figure 3A). The histogram shows the depolarization after caffeine response in all the cases, in which by pre-incubating the cells with DMF–Resv KIKO cells showed a significant difference compared to KIKO-untreated cells (Figure 3B). We then measured the increase in mROS in KIKO cells and found that both DMF alone and DMF–Resv combination effectively decreased the generation of mROS in the cardiac FA model (Figure 3C).

### Drug Testing in YG8LR FA Mice

**FIGURE 4:**
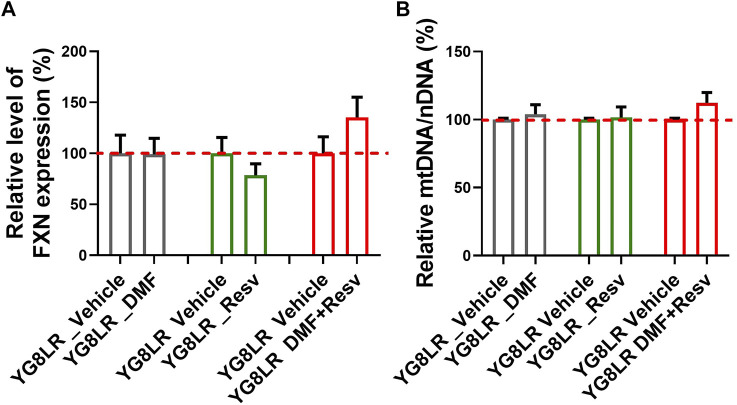
Short-term study in YG8LR mice. **(A–B)** The histogram represents the *FXN* mRNA expression and mtDNA/nDNA in brain of mice treated for 5 days with either vehicle (DMSO) or the compounds: (1) 10 mg/kg/d DMF; (2) 10 mg/kg/d Resv, or (3) 10 mg/kg/d DMF and 10 mg/kg/d Resv combined. Results were compared to YG8LR vehicle-treated mice (mean ± SD; relative levels of *FXN* %, YG8LR_DMF 99.1 ± 15.7, YG8LR_Resv 78.4 ± 11.4, YG8LR_DMF + Resv 135.3 ± 19.8, and relative mtDNA/nDNA %, YG8LR_DMF 103.98 ± 6.97, YG8LR_Resv 101.51 ± 7.8, YG8LR_DMF + Resv 112.25 ± 7.66).

To assess the *in vivo* effects of DMF, Resv, or combined DMF–Resv, groups of age- and sex-matched 2- to 4-month-old hemizygous YG8LR mice (*n* = 4–9) were treated by daily intraperitoneal injection for 5 days with either vehicle or compound: (i) 10 mg/kg/d DMF; (ii) 10 mg/kg/d Resv, or (iii) 10 mg/kg/d DMF and 10 mg/kg/d Resv combined. Brain tissue was subsequently analyzed to determine the comparative levels of *FXN* mRNA expression and mt/nDNA ratio. The results showed that in these short-term studies, there was a trend to increased levels of *FXN* mRNA expression (Figure 4A) and mt/nDNA ratio (Figure 4B) in the combined DMF–Resv treatment, although levels did not reach statistical significance. However, no increment was shown when the drugs were administered independently.

**FIGURE 5:**
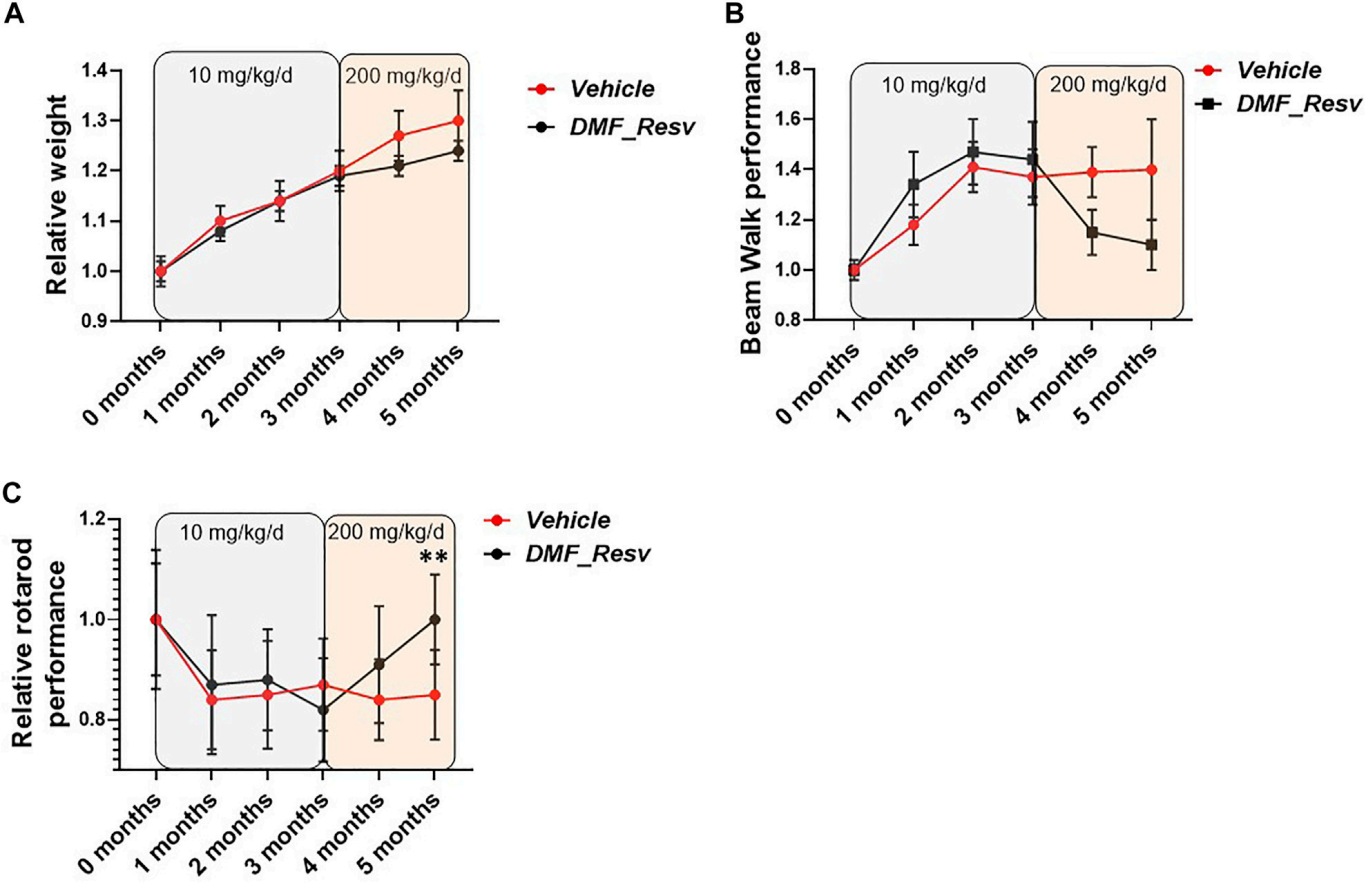
FA mice performance during the trial. YG8LR mice performances were measured compared between two groups: 1. vehicle and 2. DMF and Resv combination. **(A)** The relative weight did not show differences between treated and vehicle mice, and no differences were detected after changing the concentration of the administered drugs (from 10 mg/kg/d to 200 mg/kg/d). **(B)** The beam walk analysis showed an improvement but did not achieve statistical significance. **(C)** Interestingly, the rotarod performance showed a significant increase after the increased concentration of the drugs. Mean ± SEM, **p < 0.01 statistical significance obtained by Student’s t test analysis of 5-month data (Supplementary Table S1).

Therefore, we proceeded with long-term studies of the YG8LR mice using the combined DMF–Resv treatment approach. Groups of age- and sex-matched 2- to 3-month-old hemizygous YG8LR mice were treated by oral gavage three times per week with either vehicle (*n* = 22) or 10 mg/kg/d DMF and 10 mg/kg/d Resv combined (*n* = 21). Monthly observations were made of mouse weights and coordination abilities, as determined by performances on time taken to walk across a 1-m-long, 22-mm-wide beam and on an accelerating rotarod. After 3 months of observations, no changes were detected between the vehicle- and DMF–Resv-treated groups of mice in either average relative weight increase (Figure 5A) or beam walk tests (Figure 5B) or on the rotarod (Figure 5C). We then increased the dosage to 200 mg/kg/d DMF and 200 mg/kg/d Resv (the equivalent given to humans proportionated for mice) for a further 2 months. The results of this extended period of higher DMF–Resv dosing revealed a gradual, non-significant reduction of relative weight gain in mice treated with DMF–Resv compared to the vehicle-treated mice.

The relative beam walk performance determined by average time to cross a beam during the test period compared to the initial pretreatment average time to cross the beam (seconds) showed a trend of improvement after 3 months of treatment and at increased dosage, although no significant differences were witnessed between FA (vehicle)- and DMF–Resv-treated mice (Figure 5B). However, a trend to improved beam walk performance was detected in mice treated with DMF–Resv compared to the vehicle-treated mice throughout the three to 5-month treatment period, although the values did not achieve a statistically significant difference (Figure 5B). Interestingly, after 3 months of treatment, at 5 months of age, the DMF–Resv-treated mice showed an improvement in the relative rotarod performance, which was determined by average latency to fall during the test period compared to the initial pretreatment average latency to fall (seconds) (Figure 5C). A statistically significant increase in rotarod performance was detected in mice treated with DMF–Resv compared to the vehicle-treated mice by the 5 months time point (Figure 5C). Overall, the *in vivo* studies of YG8LR mice indicate good tolerability and positive beneficial behavioral effects of prolonged high dosing of combined DMF–Resv treatment.

## Discussion

Friedreich’s ataxia (FA) is the most common inherited ataxia, with a prevalence of ∼1—2/50,000 and a typical onset during puberty (Labuda et al., 2000). All FA patients produce a reduced amount of functional frataxin (FXN; a mitochondrial protein) (Gellera et al., 2007; Nachbauer et al., 2011; Saccà et al., 2011). Frataxin participates in iron–sulfur cluster (ISC) biosynthesis in the mitochondria (Tsai and Barondeau, 2010; Colin et al., 2013). The formation of ISCs, which are essential for electron transfer in the mitochondrial electron transport chain, is critical for optimal mitochondrial function, and we observed functional deficiency at multiple levels (Abeti et al., 2016).

There is currently no cure for FA. Nevertheless, there is an incredible ongoing worldwide effort by researchers and clinicians working on FA, alongside patients and advocacy groups, to find the most efficacious way to improve FA patients’ life.

Two main therapeutic strategies are considered for FA, and these include a. promoting an *FXN* expression increase at the gene level (Libri et al., 2014; Soragni and Gottesfeld, 2016; Abeti et al., 2018b), b. promoting downstream effects, reducing oxidant stress, and ameliorating mitochondrial function (Marmolino et al., 2010; Jasoliya et al., 2017; Lynch et al., 2018).

Our study aimed to investigate which drug and/or which combination of drugs will demonstrate to be most efficacious to increase the expression level of *FXN* mRNA, consequently improving mitochondrial health in patients’ cells and FA models’ neurons and cardiomyocytes *in vitro* and proving to ameliorate the motor skills *in vivo* in FA mouse models. We screened eight molecules that have been previously studied as potential FA treatments.

We selected NR (Bieganowski and Brenner, 2004; Chan et al., 2013), He (Napoli et al., 2007), BM (Mason et al., 2010; Giardino et al., 2013), Resv (Alcaín and Villalba, 2009; Haigis and Sinclair, 2010), EC (Qureshi et al., 2021), HDAC109 (Soragni and Gottesfeld, 2016; Rodden et al., 2021), MB (Bistas and Sanghavi, 2021), and DMF (Sahdeo et al., 2014; Jasoliya et al., 2017; Jasoliya et al., 2019). The compounds have been chosen after a more extensive screening in which they were deemed as non-toxic at the concentrations and incubating period used. By looking at the mitochondrial DNA (mtDNA) copy number, instead of nuclear DNA (nDNA), we found that BM, Resv, and DMF showed a significant increase. Instead, by measuring the *FXN* mRNA levels, a significant increase was reached by DMF. Comparing the fold change of *FXN* mRNA and mtDNA/nDNA on the same graph, we found that overall DMF and Resv seemed to be the most efficacious treatments.

We then applied these treatments on cerebellar granule neurons (CGNs) and cardiomyocytes from FA mouse models and confirmed that the mitochondrial activity was also improved under DMF or DMF–Resv treatment and the excess of mitochondrial reactive oxygen species (mROS) was decreased compared to vehicle-treated cells. However, in our *in vitro* studies, the beneficial effects appeared to be related to DMF, rather than to Resv. Subsequently, we tested the DMF–Resv combination *in vivo*, in a trial dosing the YG8LR model. Short-term studies, using a drug concentration of 10 mg/kg, showed a trend to increased levels of *FXN* mRNA expression and mt/nDNA ratio in the combined DMF–Resv treatment (not with DMF and Resv alone), although levels did not reach statistical significance and no improvement in the behavioral tests (data not shown). By changing the way of administration (oral gavage) and then increasing the concentration of the drugs to the equivalent given to humans proportionated for mice (200 mg/kg), the DMF–Resv-treated mice showed an improvement in performance on the rotarod exercise when compared to untreated (vehicle) mice. No statistical significance was witnessed in the beam walking exercise, although there was an improvement in mice treated with DMF–Resv as the time taken to walk was much shorter than the mice treated with vehicle only. The body weight remained unchanged. In summary, *in vivo* results suggest that the combination of the DMF–Resv is somehow more effective than the drugs on their own, but this is not reflected in the *in vitro* experiments on primary cells, in which DMF alone was similarly effective as the combination with Resv. It is not a surprise that *in vivo* and *in vitro* studies show different efficacy as the former is susceptible to multiple metabolic processes that are continuously taking place in the body, while the latter has less variability. This could be due to the possibility that the single drug is broken down by a number of reactions that occur continuously in the whole body, and therefore, the drug would not be effective when used directly in mice. However, this will allow us to understand how a drug or a combination of drugs works within body processes and therefore pivotal before proposing novel therapeutic strategies in humans.

Our work corroborates and supports previous studies indicating that DMF and Resv could be valid compounds to treat FA patients. Indeed, previously, we have shown that DMF alone is able to increase *FXN* levels and mitochondrial biogenesis (Jasoliya et al., 2017; Jasoliya et al., 2019). DMF is known as an inducer of the nuclear factor erythroid 2-related factor 2 (Nrf2) pathway, but it operates on both the main FA therapeutic strategies mentioned before (Jasoliya et al., 2017; Jasoliya et al., 2019). This might be the reason why it seems to be a successful candidate even in our studies. However, treatments with high dose of Resv also showed a downstream effect increasing the protection from oxidative stress and improving some of the clinical outcome measures, which include neurologic, audiologic, and speech functions (Yiu et al., 2015). In the absence of changes in *FXN* levels, Resv’s downstream effect includes the transcription of PGC-1α (through SIRT1), which is known to increase the mitochondrial biogenesis and antioxidant defenses, also enhancing the Nrf2 pathway antioxidant responses (Marmolino et al., 2010). Overall, our studies show that DMF and Resv are useful candidates to potentially alleviate the pathophysiology of FA and that *in vivo* studies are crucial to assess the potential benefit of novel drugs. Further studies will be required to prove these findings.

## Data Availability

The raw data supporting the conclusion of this article will be made available by the authors, without undue reservation.
