# Erratum: A Drug Combination Rescues Frataxin-Dependent Neural and Cardiac Pathophysiology in FA Models

**DOI:** 10.3389/fmolb.2022.968121

**Published:** 2022-07-01

**Authors:** Frontiers Production Office

**Affiliations:** Frontiers Media SA, Lausanne, Switzerland

**Keywords:** friedreich’s ataxia (FA), frataxin (FXN), dimethyl fumarate (DMF), resveratrol (Resv), mitochondrial membrane potential (ΔΨm), reactive oxygen species (ROS)

Due to a production error, the **Funding statement** for this article was omitted:

“We would like to thank the funders that supported us, in particular FARA (Bronya J. Keats International Research Collaboration Award) and an NIH grant we received (4R33NS106719-03 Pharmacodynamics and *in vivo* efficacy of fumarate for mitochondrial disease *in vivo*). Besides, we would like to acknowledge the UCLH/UCL for which PG works and receives a proportion of funding from the Department of Health's NIHR Biomedical Research Centers funding scheme, and the support given by the CRN: North Thames, National Institute for Health Research.”

The publisher apologizes for this mistake. The original version of this article has been updated.

