# Supplementary figures and images for: A Drug Combination Rescues Frataxin-Dependent Neural and Cardiac Pathophysiology in FA Models
Source: Front Mol Biosci. 2022 May 19;9:830650. doi: 10.3389/fmolb.2022.830650 (PMC9160322; doi:10.3389/fmolb.2022.830650)

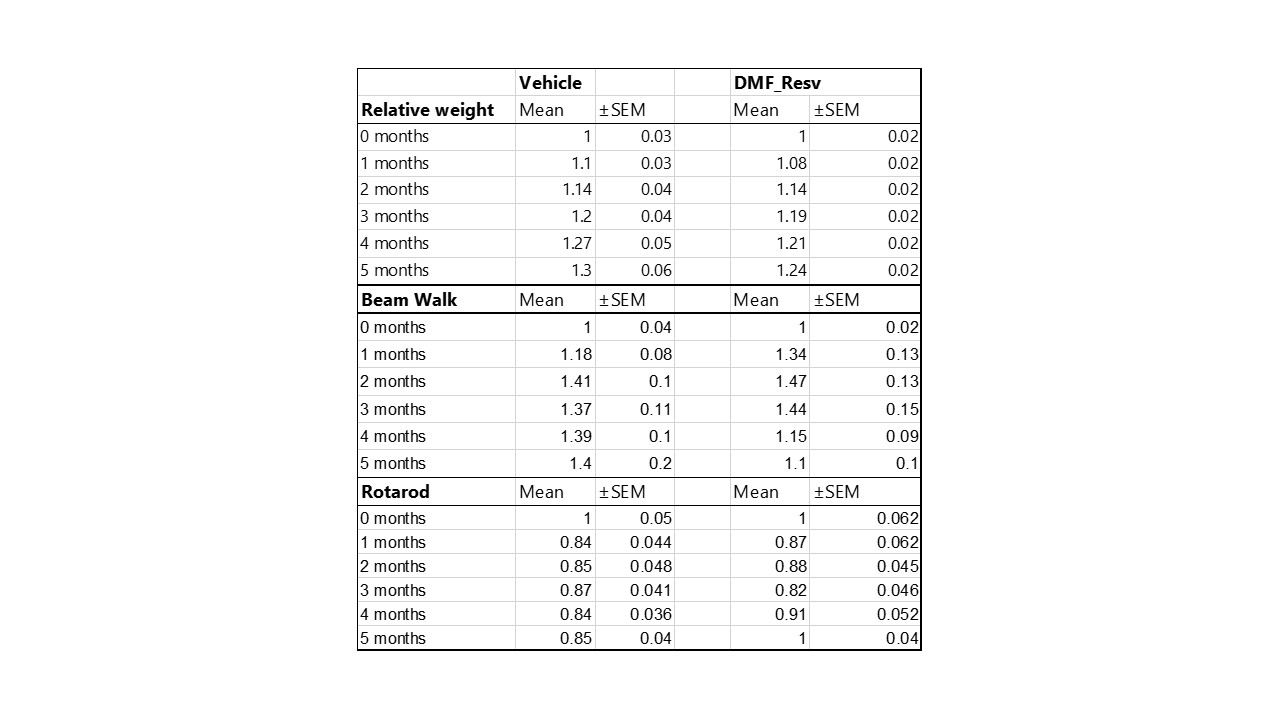

Supplement: Supplementary file 1 [file Image1.JPEG]
